# Pain intensity, disability and depression in individuals with chronic back pain^1^

**DOI:** 10.1590/0104-1169.3492.2453

**Published:** 2014

**Authors:** Márcia de Oliveira Sakamoto Silva Garbi, Priscilla Hortense, Rodrigo Ramon Falconi Gomez, Talita de Cássia Raminelli da Silva, Ana Carolina Ferreira Castanho, Fátima Aparecida Emm Faleiros Sousa

**Affiliations:** 2 MSc; 3 PhD, Adjunct Professor, Universidade Federal de São Carlos, São Carlos, SP, Brazil; 4 Undergraduate student in Psicology, Universidade Paulista, Ribeirão Preto, SP, Brazil; 5 Doctoral student, Escola de Enfermagem de Ribeirão Preto, Universidade de São Paulo, WHO Collaborating Centre for Nursing Research Development, Ribeirão Preto, SP, Brazil; 6 Specialist in Family Intervention, Professor, Universidade Paulista, Ribeirão Preto, SP, Brazil; 7 PhD, Full Professor, Escola de Enfermagem de Ribeirão Preto, Universidade de São Paulo, WHO Collaborating Centre for Nursing Research Development, Ribeirão Preto, SP, Brazil

**Keywords:** Low Back Pain, Pain Measurement, Activities of Daily Living, Depression

## Abstract

**OBJECTIVES:**

to measure the pain intensity, identify the disability and depression levels in people with chronic back pain and to correlate these variables. A cross-sectional, descriptive and exploratory study was undertaken at the Pain Treatment Clinic of the University of São Paulo at Ribeirão Preto Hospital das Clínicas, between February and June 2012, after receiving approval from the Ethics Committee at the University of São Paulo at Ribeirão Preto College of Nursing.

**METHOD:**

sixty subjects with chronic back pain participated. The instruments used were: the 11-point Numerical Category Scale, the Roland-Morris Disability Questionnaire and the Beck Depression Inventory. To analyze the data, the arithmetic means, standard deviations and Spearman's correlation coefficient were calculated.

**RESULTS:**

the findings show that the participants presented high pain, disability and depression levels. The correlation between pain intensity and disability and between pain intensity and depression was positive and weak and, between disability and depression, positive and moderate.

**CONCLUSION:**

the study variables showed moderate and weak indices and the mutual correlations were positive.

## Introduction

Pain is a multidimensional and subjective phenomenon. Therefore, individuals in pain need to be treated holistic and individually^(^ 1 ^)^. 

For the health professionals to be able to holistically manage pain, a comprehensive assessment of this individual is needed, considering physical, mental, social and spiritual aspects. Therefore, these professionals should understand that pain means an aggravation of one's existence, as not only the physical body, but life in its different dimensions is ill, including the relation with oneself, family, work and leisure^(^ 2 ^)^. 

The American Agency for Healthcare Research and Quality and the American Pain Society describe pain as the fifth vital sign, which should always be registered at the same time and in the same clinical context as the other vital signs: temperature, pulse, breathing and blood pressure^(^ 3 ^)^.

Chronic Back Pain (CBP), focused on in this study, can occur due to traumas, mechanical injury, inflammation, infection, among other, and almost all adults have already perceived it at some moment in their life. Its chronicity is defined as it continues for at least three months^(^ 4 ^)^. Its prevalence levels are high in Brazil, entailing high social and economic costs^(^ 4 ^-^ 6 ^)^. 

These costs are related to the pain-associated disabilities and to emotional problems like depression, anxiety and despair^(^ 7 ^-^ 8 ^)^. Disabilities impose limitations, such as the impossibility to develop professional activities, leave from work, changes in leisure activities and changes in family life^(^ 9 ^-^ 10 ^)^. 

Depression is the most frequent emotional condition in individuals in chronic pain and there is evidence that it is related to the pain intensity^(^ 11 ^-^ 14 ^)^. 

In that context, this study emerged as a scientific contribution proposal to better understand factors associated with CBP, such as pain intensity, disability and depression; it provides support for health professionals to better perceive the changes the CBP causes and the importance of assessing these factors for the purpose of pain management. Also, it provides support to choose more effective therapeutic approaches for individuals with CBP. 

The objectives in this study were: to measure the intensity of perceived CBP; identify the disability for activities of daily living; identify the depression levels and establish correlations among the study variables, as these can directly influence the activities of daily living and work activities, affecting the quality of life.

## Methods

### Ethical considerations

The project received approval from the Research Ethics Committee at the University of São Paulo at Ribeirão Preto College of Nursing, under opinion 1358/2011 and Letter CEP-EERP/USP - 294/2011, strictly complying with CNS/MS Resolution 466/12. All participants were invited to participate in the study and those who accepted signed the Informed Consent Form.

### Participants

The research participants were individuals under treatment for CBP. The data were collected between February and June 2012. The following inclusion criteria were used: being older than 18 years of age and having a medical diagnosis of non-oncologic CBP (pain for more than 3 months). The exclusion criteria were: individuals with inability to rationalize and changes in physical and/or cognitive functions that did not permit the data collection.

### Place

The data were collected at the Pain Treatment Clinic of the University of São Paulo Medical School Hospital das Clínicas (HCRP-USP). 

### Instruments

To achieve the objectives, a questionnaire was used that contained the items to collect sociodemographic and economic data, questions regarding the perceived pain, the Roland-Morris Disability Questionnaire^(^ 15 ^)^ and the Beck Depression Inventory^(^ 16 ^)^. The Roland-Morris Disability Questionnaire and the Beck Depression Inventory are freely accessible.

The pain intensity was assessed using the Numerical Categorical Scale (0 to 10 points), with 0 indicating "no pain", 10 "maximum pain", while the other scores, from 2 to 9, indicate intermediary perceived pain levels. 

Disability was assessed using the Roland-Morris Disability Questionnaire, which is a specific instrument to assess disability in subjects with CBP, adapted and validated for the Brazilian culture^(^ 15 ^)^. This instrument contains 24 assertions about the activities of daily living that indicate disabilities the pain provokes, using the phrase "because of my back...". The questionnaire score is calculated using the total number of questions marked as "yes", with a range from 0 to 24, in which zero corresponds to the absence of disability and 24 to severe disability, and results higher than 14 indicate significant disability^(^ 17 ^)^.

Depression was assessed using the Beck Depression Inventory (BDI), validated for the Brazilian culture. This instrument consists of 21 items scored from 0 to 3, reflecting the intensity of the symptom. The score ranges from 0 to 63 and, the higher the score, the higher the perceived depression level. For non-diagnosed samples, the guidelines are different. Scores superior to 15 are recommended to detect dysphoria and the term "depression" should only be used for individuals scoring more than 20, preferably with a concomitant clinical diagnosis^(^ 16 ^)^.

### Procedures

A cross-sectional, descriptive and exploratory research was undertaken. 

First, the subjects were selected through their medical file. The analysis of the patient histories served to identify the CBP. Next, after the participants were invited and agreed to participate, the data collection started with the use of the abovementioned instruments. 

### Analysis of the results

To analyze the sociodemographic data and the data related to pain, depression and disability, descriptive statistics were used with central trend (mean - MA, median - Med, minimum - Min and maximum - Max) and dispersion measures (standard deviation - SD), besides percentage calculations. The software Statistical Package for Social Sciences (SPSS) 18 for Windows was used.

The correlations among pain intensity, disability and depression were calculated using Spearman's correlation coefficient, which quantifies the degree of dependence between two variables, using a single figure to describe the relation among them. The significance level was set at 0.01^(^ 18 ^)^.

## Results

The participants were sixty individuals with CBP. Table 1 presents the sociodemographic distribution, showing that most participants were female (63.33%), with a mean age of 54.8 years, ranging between 22 and 91 years.

**Table 1:** Distribution of chronic back pain according to sociodemographic variables. Ribeirão Preto, SP, Brazil, 2012

Variables		Absolute figures (n)	Percentage (%)
Age			
	22-30	1	1.67
	31-40	7	11.67
	41-50	15	25
	51-60	15	25
	61-70	15	25
	71-80	5	8.32
	81-91	2	3.34
Marital status			
	Married	37	61.67
	Divorced	8	13.33
	Fixed partner	6	10
	Widowed	6	10
	Single	3	5
Education			
	Illiterate	5	8.33
	Unfinished primary education	37	61.67
	Finished primary education	3	5
	Unfinished secondary education	3	5
	Finished secondary education	9	15
	Unfinished higher education	0	0
	Finished higher education	3	5
Profession*			
	Domestic services	23	38.33
	Retired	12	20
	Services provided	5	8.33
	Others	12	33.34
Family income^† ^(MW)		2,20 (1-10,45)	

* Classification of Occupation and Activity according to IBGE, 2011

† Family income in minimum wages, calculated based on R$622, Jun/2012

Most subjects, i.e. 95% used pain medication and the mean duration of the perceived pain was 108 months.

Among the 60 participants, 23.33% were engaged in other types of pain treatments, including psychiatric, physiotherapeutic, psychological treatments, water gymnastics and gymnastics classes. The place where the subjects were being treated does not offer other types of treatment except medication.

In Table 2, the pain intensity, disability and depression scores are displayed, showing arithmetic means, standard deviations, medians, minima and maxima in the CBP patients studied.

**Table 2:** Distribution of pain intensity, disability and depression scores (mean, standard deviation, median, minimum and maximum) in CBP patients. Ribeirão Preto, SP, Brazil, 2012

	Mean	Standard deviation	Median	Minimum	Maximum
Pain intensity (0-10)	7.38	2.14	8	1	10
Disability (0-24)	19.87	2.96	21	12	24
Depression (0-63)	24.98	13.57	22.5	3	61

As observed, with regard to the results obtained in the Roland-Morris Questionnaire, 95% of the subjects scored higher than 14, indicating an important degree of disability. The results obtained through the Beck Depression Inventory showed that 61.66% of the subjects scored higher than 20, suggesting depression symptoms, as this sample was not diagnosed earlier.

The correlations were calculated using Spearman's correlation coefficient and the significance level was 0.01. The correlation found between pain intensity and disability was positive and weak (0.232). Between the variables pain intensity and depression, a weak and positive correlation was also observed (0.166). The correlation between the variables disability and depression was moderate and positive (0.362).

## Discussion

As regards the participants' perceived pain intensity, the mean score was 7.38 points and 55% of the interviewees showed a pain intensity of 8.0 points or higher; hence, it can be considered that the study subjects showed high levels of pain intensity. Some Brazilian and international authors also found CBP patients with high levels of pain intensity^(^ 7 ^,^ 9 ^,^ 19 ^-^ 20 ^)^.

As regards the disability, assessing using the RMDQ (0-24 points), the mean score was 19.87 points, suggesting that the individuals in this sample presented high levels of functional disability. In Brazil and abroad, there are studies showing similar results in CBP patients^(^ 12 ^-^ 14 ^,^ 19 ^)^.

Concerning depression, assessed using the BDI (0 to 63 points), the mean score in the study sample was 24.98 points, suggesting some level of depression. Other studies in the global literature showed similar results^(^ 7 ^,^ 9 ^,^ 14 ^,^ 19 ^,^ 21 ^)^.

The existing correlations between pain intensity and disability and between pain intensity and depression were weak and positive. Therefore, it is considered that the pain intensity weakly influences the disability and depression rates. Hence, the higher the perceived pain intensity, the greater the pain-related disability and the greater the possibilities of depression-related symptoms. The opposite cycle may also be happening. 

It is suggested that pain can cause disability and depression, as the pain can reduce movements and thus cause disability. An, in turn, disability reduces movements, causing pain, turning into a vicious circle. 

The weak correlation found between the pain intensity and disability and between the pain intensity and depression, however, suggests that, although these variables are related, disability and depression happen not only in function of the pain intensity, but other factors not studies may also contribute.

The correlation between disability and depression was positive and moderate, suggesting that, the greater the disability related to the chronic back pain, the greater the possibility of depression-related symptoms or vice-versa. Pain and disability can cause depression, as both restrict family and social life, work and leisure activities. Some Brazilian and international studies have also observed these correlations^(^ 7 ^,^ 9 ^,^ 14 ^,^ 19 ^)^.

Disability prevents people from doing activities they used to, such as housework, sports, social interaction etc., allowing depression symptoms to appear. The opposite can also be true, that is, depressed subjects tend to get more isolated and lose motivation to engage in any kind of activity.

In this and other studies mentioned below, the researchers' great concern with assessing various aspects of CBP is observed, identifying indices related to the impact of pain in daily life. This is extremely important to further understand such a complex phenomenon and, thus, to formulate pain management proposals.

Researchers have developed a study to assess perceived pain in CBP patients and compare it with quality of life and physical disability levels. They found that the mean disability score was 14.4 points, mean pain intensity 5.4 points and mean quality of life 48.1 points, showing the physical domain as the most impaired, with 44.1 points. The subjects showed high levels of pain and disability, with a strong association between the physical quality of life domain and disability and a moderate association between disability and the psychological quality of life domain^(^ 8 ^)^.

In a study, the researchers observed that depression appeared in 52% of CBP subjects, with a substantial relation between depression and CBP cases. The authors suggest that the large majority of the subjects suffering from CBP experience emotional problems like depression, anxiety and despair, besides being exposed to determining socioeconomic factors. Therefore, these components need to be identified with a view to a more complete and effective approach in the management of CBP^(^ 9 ^)^.

In a study of subjects with CBP, the researchers assessed depression using the BDI (11.86), disability using the RMDQ (12.7) and coping strategies and pain control beliefs with the help of the Coping Strategies Questionnaire e Beliefs about Pain Control Questionnaire (BPCQ-PL). Disability was positively correlated with rest, relaxation and the fact of requesting social help, that is, the greater the disability, the more the subject rests, relaxes and requests social help. In addition, depression is positively correlated with the fact of requesting social help and relaxation. Therefore, the stronger the depression, the more the subject tends to relax and request social help. It was also observed that the strategies to cope with the chronic pain are related with the pain control beliefs, depression and disability^(^ 22 ^)^. 

In another study, researchers studied the correlation between disability (Oswestry Disability Index), depression (BDI), physical activity (International Physical Activity Questionnaire), quality of life (SF-36) and sleep (Pittsburgh Sleep Quality Index) in subjects with CBP. The subjects were divided in three groups: group I (outpatient treatment), group II (preoperative) and group III (control). The results showed that all parameters in group I were lower than in group II and that all parameters in the control group were higher than in the other groups. Disability and depression were positively correlated with physical activity in group II and physical activity was correlated with physical function and vitality (subparamters of quality of life) in all groups. As observed, there was no correlation between sleep quality and physical activity in the three groups and no effect of physical activity on disability, depression and sleep quality was observed in group III^(^ 23 ^)^. 

Researchers developed a study and found a mean disability score of 8.64 points on the RMDQ in CBP patients. After assessing the results, they concluded that the individuals who felt back pain over a longer period and had low self-efficacy scores presented less effective clinical outcomes. The authors associated the back pain with psychological factors and observed that disability was associated with a negative perception about the consequences of the disease, low self-efficacy and depression^(^ 13 ^)^.

In one study, the authors found a mean pain intensity level of 7.8 points, with 80.7% of the patients showing moderate to severe disability and 36.7% depression levels. Three factors were found related to the disability: lack of paid work, low self-efficacy and depression. They also observed a higher disability prevalence among the subjects with intense pain, prolonged pain, depressive symptoms, fatigue, low self-efficacy, high levels of fear and avoidance of pain^(^ 7 ^)^. 

The researchers observed that the subjects with depression symptoms had a 1.2 times higher chance of disability than the subjects without this symptom. Therefore, treating depressive symptoms is effective to minimize the disability. Pain-related disability impairs different aspects of daily life and provokes mental suffering^(^ 7 ^)^.

In a study involving participants with CBP, the researchers observed high levels of pain intensity, disability and depression. The results showed that disability and depression revealed a weak and positive correlation, suggesting that disability increases with depression or vice-versa. As regards the physical function, it was observed that, when it decreases the pain intensity, anxiety, depression and fear increase, while the quality of life decreases when the pain intensity and fear increase^(^ 19 ^)^.

A study was conducted to examine the influence of catastrophization and other psychological variables on pain and disability in 78 subjects with CBP. They observed in the results that the CBP patients obtained a mean score of 11.6 points on the RMDQ and 5.3 points on the VAS, concluding that perceived pain and disability are strongly correlated with the catastrophization of pain, fear and depression^(^ 12 ^)^. 

In the abovementioned studies^(^ 7 ^,^ 12 ^-^ 13 ^,^ 19 ^)^, a relation is observed between disability and depression, which may suggest that individuals who are unable to accomplish their activities because of the pain feel disabled and powerless. In addition, subjects with high depression rates tend to feel more isolated and less motivated with regard to the treatment. 

Researchers undertook a study with CBP subjects and observed an association between CBP and work-related psychosocial factors. The work-related psychosocial factors were assessed using the Brief Job Stress Questionnaire (BJSQ) and the five factors that stood out were: interpersonal stress at work, satisfaction at work, depression, supervisor support at work and satisfaction in daily life. Besides the psychosocial factors, occupational ergonomics, disability to work and the family history are also related with the CBP and all of these factors can change the CBP from mild to moderate^(^ 10 ^)^. 

Individuals facing difficulties to accomplish activities of daily living and who are unable to work tend to get distanced from social life and avoid leisure activities. Social isolation and the avoidance of pain-related activities can reduce the self-efficacy and increase the chance of developing depressive and disability symptoms^(^ 7 ^)^.

In a study to analyze the relation among psychological factors, physical performance and disability, it was observed that the mean pain intensity score found was 5 points, the mean disability score (RMDQ) 12.6 points and the mean depression (BDI) score 7.3 points. No correlation was found among disability, depression and physical performance, differently from the present study^(^ 14 ^)^.

The researchers in this study raise the hypothesis that the correlation may not have existed, differently from several other studies, due to the sample characteristics, such as the subjects' mean age (38.5 years), the mean pain intensity (5) and the mean duration of the perceived pain (13 months). When comparing these data with the present study, a difference in these characteristics is observed, as the present sample shows a higher mean age (54.8 years), pain intensity (7.38) and duration of perceived pain (108 months).

Therefore, it is considered necessary to assess the physical, psychological (personal history, anxiety symptoms, depression, cognition, pain beliefs, forms of coping with the pain among others) and social components (work environment, satisfaction, stress at work, financial compensation, among others) for the professionals to understand how the individual understands and adapts to the pain. Thus, the pain can be managed in the most complete and effective way possible in order to prevent disabilities and chronicity.

Negative thoughts and fatigue are frequent symptoms in cases of depression and can interfere in the form of dealing with the pain and contribute to the presence of disability. Therefore, treating the depressive symptoms can be an effective strategy to minimize the disability and pain.

## Conclusion

Based on the results, it is observed that the study participants presented high levels of pain, disability and depression and that the pain intensity is positively correlated with disability and depression, with an even stronger positive correlation between disability and depression.

This study can contribute to picture aspects of the pain phenomenon which health professionals have not assessed yet in the management of chronic back pain. Assessing variables like disability and depression is necessary to discover individual aspects of the painful experience and, thus, propose pain management based on the reality found. These aspects are directly related with the perceived pain and losses in daily life.

These findings contribute to the understanding that pain management should be multi and inter-dimensional (emotional, social and physical), that is, all health professionals (physicians, psychologists, nurses, physical therapists, among others) should work together in search of individual care, and that pain assessment should be reconsidered in teaching and research as well. 
